# Circulating miRNAs as non-invasive biomarkers to predict aggressive prostate cancer after radical prostatectomy

**DOI:** 10.1186/s12967-019-1920-5

**Published:** 2019-05-23

**Authors:** C. Hoey, M. Ahmed, A. Fotouhi Ghiam, D. Vesprini, X. Huang, K. Commisso, A. Commisso, J. Ray, E. Fokas, D. A. Loblaw, H. H. He, S. K. Liu

**Affiliations:** 1 Biological Sciences, Sunnybrook Research Institute, Sunnybrook Health Sciences Centre, 2075 Bayview Avenue, Toronto, ON M4N 3M5 Canada; 2 Department of Medical Biophysics, University of Toronto, Toronto, Canada; 3 Princess Margaret Cancer Centre, niversity Health Network, Toronto, Canada; 4 Department of Radiation Oncology, Sunnybrook-Odette Cancer Centre, University of Toronto, Toronto, Canada; 5 Department of Radiotherapy and Oncology, Goethe-Universität Frankfurt am Main, Frankfurt, Germany

**Keywords:** Circulating biomarker, Prostate cancer, miRNA, miR-17 family

## Abstract

**Background:**

Prostate cancer is an extremely heterogeneous disease. Despite being clinically similar, some tumours are more likely to recur after surgery compared to others. Distinguishing those that need adjuvant or salvage radiotherapy will improve patient outcomes. The goal of this study was to identify circulating microRNA that could independently predict prostate cancer patient risk stratification after radical prostatectomy.

**Methods:**

Seventy-eight prostate cancer patients were recruited at the Odette Cancer Centre in Sunnybrook Health Sciences Centre. All patients had previously undergone radical prostatectomy. Blood samples were collected simultaneously for PSA testing and miRNA analysis using NanoString nCounter technology. Of the 78 samples, 75 had acceptable miRNA quantity and quality. Patients were stratified into high- and low-risk categories based on Gleason score, pathological T stage, surgical margin status, and diagnostic PSA: patients with Gleason ≥ 8; pT3a and positive margin; pT3b and any margin; or diagnostic PSA > 20 µg/mL were classified as high-risk (n = 44) and all other patients were classified as low-risk (n = 31).

**Results:**

Using our patient dataset, we identified a four-miRNA signature (miR-17, miR-20a, miR-20b, miR-106a) that can distinguish high- and low-risk patients, in addition to their pathological tumour stage. High expression of these miRNAs is associated with shorter time to biochemical recurrence in the TCGA dataset. These miRNAs confer an aggressive phenotype upon overexpression in vitro.

**Conclusions:**

This proof-of-principle report highlights the potential of circulating miRNAs to independently predict risk stratification of prostate cancer patients after radical prostatectomy.

**Electronic supplementary material:**

The online version of this article (10.1186/s12967-019-1920-5) contains supplementary material, which is available to authorized users.

## Background

Prostate cancer is the second most commonly diagnosed cancer and a leading cause of cancer-related mortality in men worldwide [1–3]. Despite possessing similar clinicopathological features, some prostate cancer patients are at high risk of developing local and/or distant recurrence and succumbing to their disease, whereas many others will have clinically indolent disease and will not benefit from further intervention. Determining which patients are unlikely to derive a therapeutic benefit from radiation treatment after surgery will prevent overtreatment, removing the burden of unnecessary therapy and side-effects from patients and healthcare systems. Conversely, the ability to accurately identify which patients harbour residual aggressive disease that require more intensive therapy (i.e., dose intensification or early integration of systemic therapies) could improve patient outcomes.

It has been estimated that upwards of 30% of patients will develop biochemical recurrence (BCR) after radical prostatectomy [4–6]. High-risk features for BCR include high Gleason score, high pathological T score, extraprostatic extension, seminal vesicle invasion, positive margins, and rapid prostate-specific antigen (PSA) doubling time [7–9]. Adjuvant radiotherapy can reduce the risk of BCR by approximately 50%, but is associated with higher incidence of acute and late normal tissue toxicity [10]. Unfortunately, we are currently limited by the existing assays to accurately predict high-risk prostate cancer BCR. Although PSA doubling time is used for monitoring recurrence after radial prostatectomy, it is not able to determine in advance which patients will recur or are at a high risk of recurrence in the future. Furthermore, PSA does not provide information on which patients may benefit from adjuvant or salvage therapy. In the post-radical prostatectomy setting, Decipher [11, 12] is the one commercially available genomic test to combat the limitations of PSA. It uses surgical tissue to predict disease aggressiveness and the probability of progression after radical prostatectomy. Although this test is very valuable to guide treatment decisions, it cannot be used to monitor treatment response and disease progression over time, as samples are taken from a single timepoint. Furthermore, only the index lesion is macrodissected for Decipher analysis, which does not take into consideration the extensive heterogeneity within a tumour [11]. Therefore, there is a significant clinical need to find non-invasive biomarkers to identify patients at a high risk of recurrence, monitor their disease progression and treatment response, and optimize their personalized treatment regimens. There is recent evidence that liquid biopsies (biomarkers found in patient biofluids, i.e. blood and urine) are likely to be more representative of the whole tumour’s genomic landscape compared to tumour sampling [13, 14].

Using blood collected from patients after radical prostatectomy, we profiled the expression of circulating miRNAs (miRNA, miR) and identified a signature of four microRNAs that predict for high-risk prostate cancer: hsa-miR-17-5p, hsa-miR-20a-5p, hsa-miR-20b-5p, hsa-miR-106a-5p (henceforth referred to as miR-17, miR-20a, miR-20b, miR-106a, respectively). Intriguingly, these four miRNAs are all from the same miRNA family, which describes miRNA that contain the same seed sequence and should therefore theoretically target the same downstream pathways. Our research expands the current knowledge of these four members of the oncogenic miR-17 family by describing them as circulating biomarkers that can independently predict for high-risk prostate cancer after radical prostatectomy.

## Materials and methods

### Patient selection

Patients who had previously received radical prostatectomy and were referred for postoperative radiotherapy to a multidisciplinary genitourinary clinic at the Odette Cancer Centre in Sunnybrook Health Sciences Centre were prospectively recruited and consented. Patients were recruited using an institutional Research Ethics Board-approved protocol. The protocol and methods were approved by the Sunnybrook Research Ethics Board (REB# 035-2015), and the study was carried out in accordance with institutional guidelines. Informed consent was obtained from all patients. Patient characteristics and tumour features were collected and analyzed. A total of 78 male patients who had previously undergone radical prostatectomy were selected.

### Risk stratification

Patients were classified into low-risk and high-risk categories based on pathological T stage, Gleason Score, diagnostic PSA level and margin status, which were all obtained from patients’ surgical pathology report (Additional file 1: Table S1). Criteria for high-risk prostate cancer is as follows: pT3a and positive margin; pT3b any margin; Gleason 8 and above; or diagnostic PSA greater than 20 ng/mL. All other patients were considered low-risk.

### Sample processing and miRNA isolation

Blood samples were obtained simultaneously for PSA measurement and serum miRNA extraction. Serum was collected within 2 h of blood draw. Mean and median time after radical prostatectomy to sample collection was 31 and 9 months, respectively (range = 3 weeks to 16 years). Circulating miRNAs were collected from serum using the Plasma/Serum Circulating and Exosomal RNA Purification Mini Kit (Norgen Biotek Corp., Canada).

### miRNA expression analysis

miRNA expression was performed using the NanoString nCounter technology. NanoString output was normalized using the R package NanoStringNorm (version 1.2.1) with the parameters CodeCount = “geo.mean”, Background = “mean” and SampleContent = “housekeeping.geo.mean”. After normalizing the expression, three samples were removed from downstream analysis due to insufficient RNA quantity and/or quality. Abundant miRNAs were selected by filtering out any miRNAs with zero expression in more than 10% samples. All samples had expression values for at least 90% miRNAs.

Upon normalization, the count matrix where each row is a miRNA and each column is a sample was subjected to negative binomial test for differential expression analysis. This was done using the R package DESeq [15]. The sizeFactors() function of DESeq was set to zero to avoid over-normalization before estimating dispersion using the function estimateDispersions(). The nbinomTest() function was used to compute the differential expression between two groups of patients—either for high-risk versus low-risk, high Gleason score versus low Gleason score, etc. The fold change cutoff was set to 1.5 fold and *p* value cutoff was set to 0.05 across the study if not otherwise specified. The plots were generated using the R package ggplot2.

### TCGA data analysis

The miRNA-seq data from TCGA dataset were downloaded from the GDC portal. The clinical data for BCR-free survival analysis were downloaded from the cBioPortal. The survival analysis was performed using the R package Survival and the Kaplan–Meier plots were generated using the R package survminer.

### Cell lines and cell culture

PC3 prostate cell line was purchased from the American Type Culture Collection (ATCC, USA). Cells were maintained in Dulbecco’s Modified Eagle Medium (DMEM) with 4.5 g/L glucose (Invitrogen), and supplemented with 10% FBS (Invitrogen) and penicillin (100 U/mL)—streptomycin (100 μg/mL) (Invitrogen). Cells were maintained in a humidified 37 °C incubator with 5% CO_2_. Cells were passaged upon 70–90% confluency and tested regularly with MycoAlert™ Mycoplasma Detection Kit (Lonza).

### Transfection of miRNA mimics

Cells were seeded into 6-well plates at a density of 2 × 10^5^ cells per well. The following day, miRNA mimics (5 μM) (Shanghai GenePharma Co. Shanghai, China; Additional file 2: Table S2) were transiently transfected into cells using Lipofectamine 2000 (Invitrogen, Canada) and Opti-MEM I (1X) reduced serum media (Invitrogen), as per manufacturer’s instructions. miRNA overexpression with mimic was verified with qRT-PCR (Additional file 3: Figure S1). Cells were collected at 24 h after transfection for proliferation, soft agar, and clonogenic assays.

### Cell proliferation assay

Transfected cells were seeded in triplicate in 6-well plates at a density of 5 × 10^5^ cells/well. Four days later, cells were trypsinized and the total number of viable cells was determined using the Countess automated cell counter (Life Technologies). This experiment was performed in three biological replicates.

### Soft agar colony formation assay

Transfected cells were resuspended in DMEM with 0.5% Agar-A (Bio Basic Inc.) and 1000 cells were seeded into 24-well plate with 0.8% Agar-A coated wells, to prevent cells from adhering to plate surface, and placed in a humidified incubator at 37 °C. Colonies were counted approximately 6 weeks later and averaged among three biological replicates.

### Clonogenic survival assay after radiation

Control and miRNA mimic–transfected PC3 cells were seeded in triplicate at the following density per well of a 6-well plate: 0 Gy—250 cells, 2 Gy—500 cells, 4 Gy—2000 cells, 6 Gy—4000 cells, and 8 Gy—6000 cells. Cells were irradiated 4 h after seeding and returned to 37 °C incubator. At day 11 after irradiation, cells were stained with crystal violet stain (0.5% crystal violet (Sigma Alderich, Canada), 25% methanol). The number of colonies (> 50 cells) were counted and surviving fraction was determined based on plating efficiency of IR-treated cells relative to mock-radiated cells. Clonogenic survival was represented in dose–response curves by fitting relative surviving fraction to the linear quadratic formula equation *S *=* e*^−*αD*−*βD*2^ using GraphPad Prism 5.0 (GraphPad Software Inc., USA), where S is the surviving fraction, α and β are inactivation constants, and D is the radiation dose in Gy.

### Statistical analysis

Statistical tests and data representation were performed using GraphPad Prism v5.01 software (GraphPad Software) for in vitro experiments. Data are represented as mean values ± standard error of the mean (SEM) unless otherwise mentioned. Statistical significance defined as *p* < 0.05.

## Results

### MicroRNA profiling

**Fig. 1:**
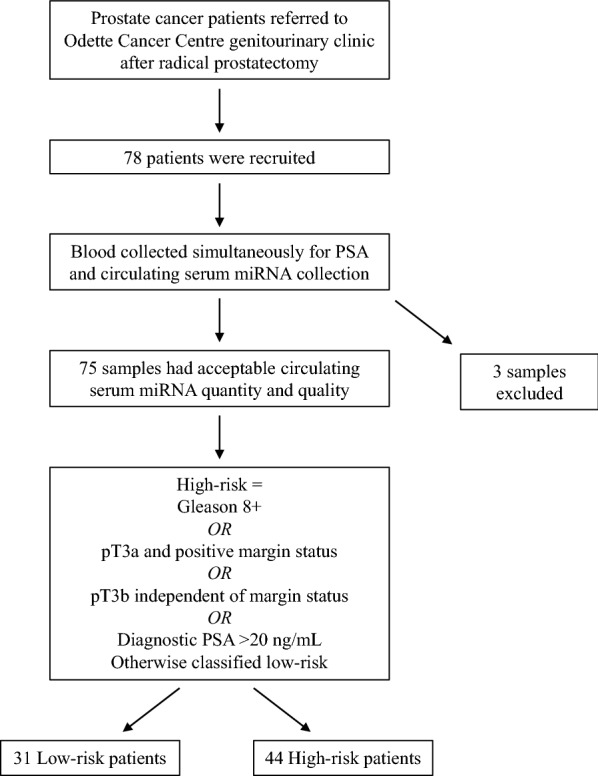
Patient sample collection workflow. Seventy-eight post-radical prostatectomy prostate cancer patients who were referred to a genitourinary clinic at Odette Cancer Centre were prospectively recruited. Blood for PSA monitoring and isolation of circulating miRNAs was collected simultaneously (median time of 9 months after radical prostatectomy). Seventy-five serum samples, yielding acceptable quantity and quality, were used for downstream NanoString nCounter analysis. Gleason score, pathological T stage, margin status, and diagnostic PSA level were used to stratify patients into low-risk (n = 31) and high-risk (n = 44) categories

**Fig. 2:**
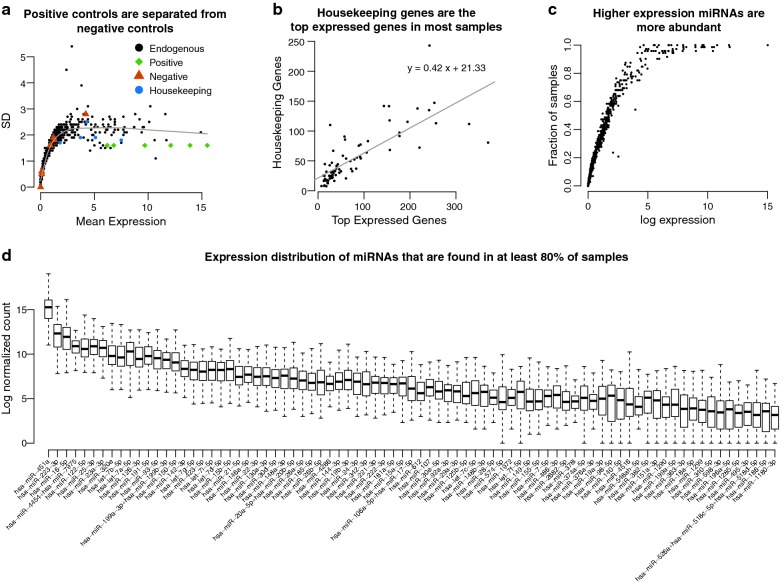
NanoString analysis of circulating miRNAs. **a** Mean (± SD) expression of all miRNAs in the library. A best fit loess curve using all data is shown in grey. **b** Estimates of RNA content, where each point represents a sample. The x-axis is the RNA estimate for each sample based on the geometric mean of the top 75 expressed miRNAs, and the y-axis is the RNA estimate based on the geometric mean housekeeping mRNA genes. **c** Expression of each miRNA and fraction of samples that each miRNA is expressed in. **d** Expression levels of miRNAs expressed in at least 80% of samples

The primary objective of this study was to identify circulating miRNAs in the post-radical prostatectomy setting that could be used to independently predict risk stratification. To identify such miRNAs, of 78 patients, 75 samples had acceptable miRNA quality and quantity. We stratified these 75 patients into high-risk (n = 44) and low-risk (n = 31) categories based on their surgical pathology (i.e. Gleason score, pathological T stage, and margin status) and diagnostic PSA (Fig. 1). Patient characteristics can be found in Table 1. Criteria for high-risk prostate cancer is as follows: pT3a and positive margin, OR pT3b any margin, OR Gleason 8 and above, OR diagnostic PSA greater than 20 ng/mL, based on the three well-defined poor prognostic groups [16]. All other patients were classified as low-risk. We profiled the expression of 828 miRNAs using NanoString nCounter technology. The positive controls in the library have higher mean expression than the negative controls (Fig. 2a). We further examined the expression of the housekeeping genes and observed that the housekeeping genes constitute the majority of the highest expressed genes in each sample (Fig. 2b). The majority of these 828 miRNAs were found only in a fraction of patients, while 81 miRNAs were expressed in at least 80% of the samples (Fig. 2c, d). A miRNA with good biomarker potential would ideally be detectable in most samples; hence we only retained these 81 miRNAs with high expression for subsequent analyses.

**Table 1:** Patient demographics

Characteristic	Low risk (n = 31)	High risk (n = 44)	Total (n = 75)
Age at surgery			
Median years (range)	61 (45–76)	64 (44–77)	63 (44–77)
PSA at diagnosis			
Median No. (range)	6.6 (4–16.7)	10 (1.8–140)	8.5 (1.8–140)
PSA at day biobanked			
Median No. (range)	0.1 (0–1.7)	0.1 (0–16.6)	0.11 (0–16.6)
No. Gleason score (%)			
6	3 (10)	0	3 (4)
7	28 (90)	19 (43)	47 (63)
8	0	9 (20)	9 (12)
9	0	16 (36)	16 (21)
No. pathological tumour stage (%)			
≤ pT2	23 (74)	3 (7)	26 (35)
pT3a	8 (26)	22 (50)	30 (40)
pT3b	0	18 (41)	18 (24)
pT4	0	1 (2)	1 (1)
No. margin status (%)			
Positive	11 (35)	30 (68)	41 (55)
Negative	20 (65)	14 (32)	34 (45)
No. salvage therapy (%)			
Radiation	23 (74)	33 (75)	56 (75)
Hormone	16 (52)	34 (77)	50 (67)

### Predictive effect of miR-17, miR-20a, miR-20b, miR-106a

**Fig. 3:**
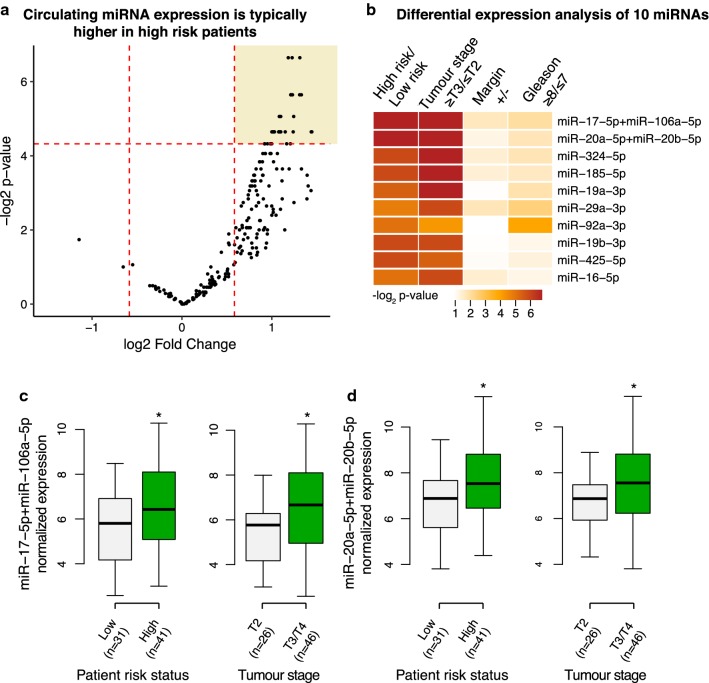
Differential expression analysis of miRNAs. **a** Volcano plot of circulating miRNAs differentially expressed in high-risk versus low-risk patient groups. The vertical red lines indicate 1.5 fold expression change, horizontal lines indicate the p-value of 0.05 in -log2 scale. **b** Differential expression analysis of top 10 miRNAs tested independently in contrasts indicated for each column. **c**, **d** Expression of miRNAs in patients with high risk or low risk and in patients with T2 or T3/T4 pathological T stage of tumour (*p *< 0.05)

In order to identify miRNAs that are differentially expressed in high versus low risk patients, we performed negative binomial test between the two patient groups. Among the 81 highly expressed miRNAs, 32 were found to be significantly upregulated (*p*-value ≤ 0.05 and fold change ≥ 1.5x) in high-risk patients compared to low-risk patients (Fig. 3a; Additional file 4: Table S3). While all of these 32 miRNAs warrant further investigation, we ranked the most important ones by estimating their differential expression between patients grouped by pathological T stage, margin status and Gleason score independently and cumulating their statistical probability values (Fig. 3b). The two top-ranked miRNAs are miR-17 + miR-106a and miR-20a + miR-20b. Among these four miRNAs, miR-17, -20a, and -106a were previously reported to be enriched in prostate carcinoma samples compared to normal prostate tissue [17]. Consistently, these miRNAs also show higher expression in the blood of patients with pathological stage pT ≥ 3 (cancer spread beyond prostate) compared to pT ≤ 2 (cancer confined to prostate) (*p *= 0.01, negative binomial test, Fig. 3c, d). pT ≥ 3 is associated with disease progression, metastasis, and decreased survival. Indeed, it is intriguing that even after all clinical evidence of tumour tissue is removed, circulating miRNAs in the blood can stratify patients into high- and low-risk categories of recurrence. Similarly, others have found that circulating miRNAs are representative of tumour biology in the adjuvant setting in breast and colon cancer [18, 19].

**Fig. 4:**
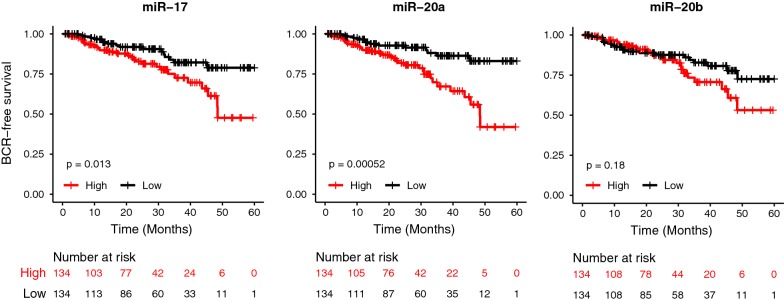
miR-17 and miR-20a are associated with biochemical recurrence in TCGA dataset. Kaplan–Meier curves of time to biochemical recurrence (BCR) after radical prostatectomy. High miR-17 and miR-20a expression is associated with significantly lower BCR-free survival (268 tumours; miR-17, *p *= 0.01, miR-20a, *p *= 0.0005; logrank test). High miR-20b expression showed a trend towards lower BCR-free survival, however this did not reach significance (268 tumours; *p *= 0.18; logrank test)

As we did not have access to the radical prostatectomy specimens, we utilized The Cancer Genome Atlas (TCGA) dataset as a surrogate to evaluate the prognostic significance of these miRNAs. We discovered that high expression of miR-17 and 20a are significantly associated with biochemical free survival at 5 years (miR-17, *p *= 0.01, miR-20a, *p *= 0.0005, logrank test, Fig. 4). We previously reported that elevated miR-106a expression is associated with shorter time to BCR at 5 years after radical prostatectomy [20]. High expression of miR-20b showed a trend towards earlier time to BCR, although this was not statistically significant (miR-20b, *p *= 0.18, logrank test, Fig. 4). Interestingly, for miR-17 and -20a, we observed an association between their expression levels and Gleason score, although an association was not seen with pathological T stage (Additional file 5: Figure S2a,b). This was also seen with miR-106a, which is known to be enriched in higher Gleason score prostate tumours [20], but not for pathological T stage (data not shown).

### Functional characterization of miRNAs in prostate cancer

**Fig. 5:**
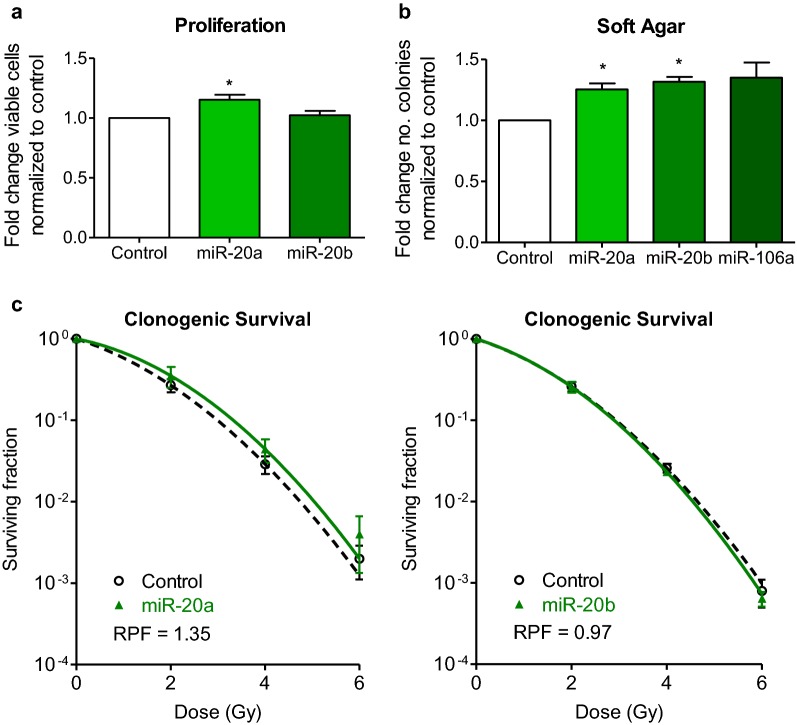
In vitro characterization of miRNAs. PC3 prostate carcinoma cells were transfected with control, miR-20a or miR-20b mimics. **a** miR-20a overexpression increased the number of viable cells (p = 0.03, paired Student’s t test). miR-20b showed no significant differences in cell viability (p = 0.5915, paired Student’s t test). **b** Soft Agar assay evaluating colony formation in a 3D matrix showed increased colony formation with miR-20a mimic (p = 0.03, paired Student’s t test) and miR-20b mimic (p = 0.01, paired Student’s t test). miR-106a overexpression also increased colony formation, however this did not reach statistical significance (p = 0.11, paired Student’s t test). **c** Radiation protection factor (RPF) is shown for each miRNA compared to respective control

The molecular underpinnings of a tumour set the stage for treatment response and disease progression. Proliferation is a key property of tumour aggression and recurrence. miR-17 has previously been described as an oncomiR (miRNA that facilitates oncogenesis), based upon functional characterization employing in vitro and in vivo models of prostate cancer, where its overexpression increases proliferation, soft agar colony formation, and tumour growth in a mouse model [21]. miR-106a was recently characterized by our group, and similar to miR-17, demonstrated increased proliferation in vitro, tumour growth in vivo [20], and resistance to radiotherapy. To the best of our knowledge, miR-20a and miR-20b have not been functionally characterized in prostate cancer. As such, we performed transient transfection experiments with miRNA mimics, and discovered that miR-20a increases proliferation in the PC3 cell line (*p *= 0.03, paired Student’s *t* test, Fig. 5a). miR-20b overexpression had no effect (*p *= 0.59, Fig. 5a).

Another factor imperative to tumour aggression is the ability to form colonies in the absence of adhering to a basement membrane. Anchorage-independent growth is indicative of tumorigenicity and is assessed by suspending cells in a 3D agar matrix. We saw increased colony formation with overexpression of miR-20a (*p *= 0.03, paired Student’s *t* test) and miR-20b (*p *= 0.01, paired Student’s *t* test), and a trend towards increased colony formation with miR-106a overexpression, although this did not reach statistical significance (*p *= 0.11, paired Student’s *t* test, Fig. 5b).

Following radical prostatectomy, radiation treatment is often used in the adjuvant or salvage setting. We have previously shown that miR-106a confers radioresistance by increasing proliferation and decreasing cell death after radiotherapy in prostate cancer models [20]. However, to the best of our knowledge, the effect of miR-20a, and miR-20b on radiation survival has not been evaluated. As such, we investigated the effect of these miRNAs on clonogenic survival. The clonogenic survival assay is the gold standard assay for cell survival after radiation treatment. miRNA mimics were introduced into PC3 cells to overexpress miR-20a and miR-20b. We found that miR-20a overexpression increased clonogenic survival in cells lines with a radiation protection factor (RPF) of 1.35, and miR-20b did not affect clonogenic survival in PC3 with an RPF = 0.97 (Fig. 5c). Future studies may highlight a use for these miRNAs as predictive biomarkers for treatment response.

Together, the function of these miRNAs can increase proliferation, colony formation, and survival following radiation treatment. This suggests that miR-17, -20a, -20b, and -106a collectively contribute to an aggressive phenotype.

## Discussion

Although patients can possess similar clinicopathological features, a subset of patients are at a high risk of cancer recurring after radical prostatectomy, whereas many others will have clinically insignificant disease. There is a need for non-invasive tests that correlate with histopathological data to better identify these patients who are at risk of recurrence.

To address this gap, we identified four circulating miRNAs that stratify prostate cancer patients after radical prostatectomy. We found that high expression of miR-17, -20a, -20b, and -106a predict for high-risk disease and high pathological T stage (pT3 and above). Furthermore, in combination with previously published works and our new data, we found that these miRNAs confer an aggressive phenotype as demonstrated utilizing in vitro validation studies, providing biological evidence to support their role in high-risk prostate cancer. Our results were consistent with analysis from the TCGA dataset where miR-17, miR-20a, and miR-106a [20] were associated with shorter time to BCR. This suggests that these miRNAs not only accurately stratify patients into risk categories, but also serve as promising candidates for prognostic biomarkers.

Our results are supported by previous studies. As previously discussed, miR-17 has been described as an oncomiR in prostate cancer [21]. miR-20a has previously been identified as a plasma biomarker that could accurately distinguish high- versus low-risk disease in treatment naïve prostate cancer patients using the D’Amico risk classification [22]. miR-20a was significantly overexpressed in patients with high-risk Cancer of the Prostate Risk Assessment (CAPRA) score, and in patients with pathological T stage 3/4 compared to T stage 1/2 [22]. Another study found that miR-20a was enriched in high/intermediate-grade prostate cancer (Gleason 7–10) compared to low-grade prostate cancer (Gleason 6 and below) [23]. Our group previously described miR-106a as an oncomiR that is associated with high-grade prostate cancer and confers an aggressive phenotype in vitro and in vivo [20]. We found that miR-106a is not only overexpressed in prostate cancer tissue compared to normal tissue but is overexpressed in high-grade tumours (Gleason 8–10) compared to low-grade tumours (Gleason 6–7). We also previously found that high miR-106a predicts for shorter time to recurrence after radical prostatectomy, which supports our results in this study. Furthermore, miR-106a has previously been described by Alhasan et al. [24] as a five-miRNA serum biomarker signature to identify patients with treatment-naïve high-risk prostate cancer.

Although there are various studies identifying prostate cancer circulating biomarkers for prostate cancer diagnosis [25–28] and treatment-naïve disease progression [24, 29, 30], there are few in the post-radical prostatectomy setting. There are numerous tissue-based miRNAs that have been shown to predict for prostate cancer biochemical recurrence after radical prostatectomy [31–35]. However, these biomarker signatures are hindered by their use of radical prostatectomy tissue, which cannot monitor tumour progression over time like circulating biomarkers.

miRNAs are small, non-coding RNA that regulate the expression of mRNA by inhibiting translation and facilitating target mRNA degradation. miRNAs have been implicated in many pathological states but are especially known for their role in cancer. In prostate cancer in particular, circulating miRNAs have been described to have diagnostic, predictive, and prognostic capabilities [36, 37]. miR-1290 and miR-375 were identified from blood samples as prognostic markers that correlated with overall survival in castrate-resistant prostate cancer [38]. miR-1246 has also been identified as a prognostic biomarker for prostate cancer; its expression correlated with pathological grade, positive metastasis, poor prognosis, and tumour aggression in vitro and in vivo [39].

Circulating miRNAs are uniquely helpful for stratification and they have intriguing qualities that would make them particularly promising biomarkers. Circulating miRNAs are non-invasive, as they can be extracted from a simple blood test at various timepoints. They are highly stable in the blood and during laboratory processing [40, 41]. Circulating miRNAs, in particular exosomal miRNAs, are thought to be involved in various aspects of tumour maintenance and progression, including local proliferation, invasion and treatment resistance; recruitment and activation of tumour-promoting immune cells; and formation of pre-metastatic niche at distant locations [42]. Specifically, exosomal miRNAs have been shown to be largely representative of cellular miRNA contents [43], and exosomes may be selectively secreted from cancer cells [44]. It was recently found that circulating tumour DNA (ctDNA) is detectable in men with de novo metastatic castrate-sensitive prostate cancer, and ctDNA was significantly reduced after ADT [45]. Furthermore, somatic mutations in ctDNA were found to be highly consistent (83%) with those in the matched prostate biopsy specimen, suggesting that ctDNA can provide a non-invasive method to identify important tumour alterations.

Although our signature can predict for the presence of high-risk disease, our clinical follow-up is not yet mature enough to establish its impact on predicting BCR (mean and median time to follow-up is 5.3 and 3.7 years after radical prostatectomy, respectively). We expect that high expression of these miRNAs will translate to increased biochemical failure, given that this signature predicts for high-risk disease. Furthermore, using the TCGA dataset as a surrogate, we did find that high expression of miR-17, -20a, and -106a [20] correlate with shorter time to BCR. However, future studies will need to determine whether this signature provides additional prognostic benefits to current clinicopathological protocols and be validated in larger multi-institutional datasets.

Furthermore, we did not have access to preoperative circulating miRNA levels for these patients. This would provide additional clinical utility by identifying a miRNA profile to distinguish men who could be spared radical prostatectomy and should undergo upfront radiation therapy instead.

A limitation of this study is that due to the small sample size we are unable to accurately assess the predictive power, which is dependent on sample size. Future studies should assess the predictive power of these miRNAs using a larger patient cohort.

Another limitation is that we did not have access to the radical prostatectomy specimens to correlate tissue miRNA expression with blood expression. However, as a surrogate analysis, we mined the TCGA and showed that these miRNAs are enriched in high-grade prostate cancer compared to low-grade prostate cancer to validate our findings.

The final limitation of this study is that the NanoString technology was unable to differentiate between miR-17 and miR-106a, as well as miR-20a and miR-20b. We therefore cannot further establish their individual contributions.

During the course of this study, other groups have developed tests to identify patients at high-risk of recurrence and disease progression after radical prostatectomy. OncotypeDx (San Diego, USA) developed Decipher—a prognostic test to identify patients’ aggressive prostate cancer that is likely to metastasize after radical prostatectomy. Decipher captures a single timepoint of the disease using radical prostatectomy tissue. An advantage of our study is that a liquid biopsy allows for serial monitoring of disease progression. With the ever-changing tumoral landscape, serial monitoring is an important feature of a biomarker. Future studies should evaluate the utility of serial timepoint collections to delineate whether circulating miRNA expression is correlated with disease progression and treatment response.

Despite these limitations, our study has significant novel data showing proof-of-principle that circulating miRNAs are detectable after radical prostatectomy, where the entire prostate has been surgically removed, and can accurately stratify patients into high- and low-risk categories. These results are hypothesis-driving, suggesting that high expression of these four miRNAs in patient blood samples is indicative of high-risk disease that is likely to recur after radical prostatectomy. Future studies will confirm this hypothesis and reveal the clinical utility of these miRNAs as predictive or prognostic biomarkers.

## Conclusion

In this study we identified that a non-invasive liquid biopsy using circulating miRNAs can accurately stratify prostate cancer patients after radical prostatectomy. Furthermore, we show that we can in fact detect circulating miRNAs after removal of the entire prostate gland, and that their expression has promising clinical utility. With future studies looking at BCR, disease progression and survival outcomes, these miRNAs could prove to be biomarkers that yield important information of a patient’s disease progression, and guide subsequent treatment in the adjuvant or salvage setting.

## Additional files

**Additional file 1: Table S1.** Individual patient characteristics and corresponding stratification into low- and high-risk groups using PSA at diagnosis, Gleason score, T stage, and margin status.

**Additional file 2: Table S2.** miRNA mimic sequences for control, miR-17, miR-20a, miR-20b and miR-106a that were used in in vitro validation studies. Sequences provided in 5’ – 3’ orientation.

**Additional file 3: Figure S1.** Representative qRT-PCR analysis for miR-20a, miR-20b and miR-106a expression in PC3 cells after miRNA mimic transfection. miRNA expression is normalized to endogenous control, SNORD.

**Additional file 4: Table S3.** Differential expression analysis of circulating serum miRNAs in low-risk vs high-risk patients. padj, p-value corrected for multiple testing using Bonferroni method.

**Additional file 5: Figure S2.** Expression of miR-17, miR-20a and miR-106a in patients with **a** Gleason score and **b** pathological T stage using the TCGA PRAD miRNA-seq dataset. Each box denotes the quartiles of the expression across samples. The black bars inside the boxes indicate the median expression of each miRNA.

## Data Availability

The datasets analysed in this study are available from the corresponding author on reasonable request.

## Abbreviations

ATCC: American Type Culture Collection
BCR: biochemical recurrence
CAPRA: Cancer of the Prostate Risk Assessment
ctDNA: circulating tumour DNA
DMEM: Dulbecco’s Modified Eagle Medium
miRNA, miR: MicroRNA
PSA: prostate-specific antigen
RPF: radiation protection factor
REB: Research Ethics Board
SEM: standard error of the mean
TCGA: The Cancer Genome Atlas
