# Supplementary material for: Circulating miRNAs as non-invasive biomarkers to predict aggressive prostate cancer after radical prostatectomy
Source: J Transl Med. 2019 May 23;17:173. doi: 10.1186/s12967-019-1920-5 (PMC6533745; doi:10.1186/s12967-019-1920-5)
Supplement: Supplementary file 1 — Additional file 1: Table S1. Individual patient characteristics and corresponding stratification into low- and high-risk groups using PSA at diagnosis, Gleason score, T stage, and margin status. [file 12967_2019_1920_MOESM1_ESM.pdf]

P #	PSA at diagnosis	PSA Post-RP	GS Primary	GS Secondary	Gleason	T Stage	Margin	Stratification	Notes
**P5004**		0.17	4	4	8	T3b	-	High Risk	
**P5006**	4	0.36	3	4	7	T3a	-	Low Risk	
**P5007**	10	0.26	4	3	7	T3a	-	Low Risk	
**P5008**	10	0.12	4	3	7	T2c	-	Low Risk	
**P5010**	18	0.04	3	4	7	T3b	-	High Risk	
**P5011**	5	0.03	4	4	8	T3a	+	High Risk	
**P5013**	6	0.02	4	3	7	T3a	+	High Risk	
**P5014**	17	0.48	4	5	9	T3a	+	High Risk	
**P5015**	140	0.03	3	4	7	T3a	+	High Risk	
**P5018**	17	0.20	4	3	7	T3a	+	High Risk	
**P5019**	17	0.01	4	3	7	T2c	+	Low Risk	
**P5021**	9	0.35	3	4	7	T3a	+	High Risk	
**P5022**	19	2.28	4	5	9	T3b	-	High Risk	
**P5026**	6	0.13	3	4	7	T2c	+	Low Risk	
**P5027**	9	0.01	4	3	7	T3b	-	High Risk	
**P5028**	7	0.02	4	5	9	T3a	-	High Risk	
**P5029**	7	0.00	4	5	9	T3a	-	High Risk	
**P5030**	7	0.04	4	3	7	T2c	+	Low Risk	
**P5031**	22	0.10	4	5	9	T3b	+	High Risk	
**P5034**	10	0.59	3	4	7	T3a	-	Low Risk	
**P5035**	20	0.99	4	5	9	T3b	+	High Risk	
**P5036**	5	0.11	5	3	8	T3a	-	High Risk	
**P5037**	11	0.10	4	3	7	T3b	+	High Risk	
**P5039**	5	0.24	3	4	7	T3b	+	High Risk	
**P5048**	5	0.16	4	4	8	T3a	+	High Risk	
**P5049**	67	16.59	5	4	9	T3b	+	High Risk	
**P5051**		0.02	3	4	7	T2c	-	Low Risk	
**P5054**	5	0.10	4	4	8	T3a	-	High Risk	
**P5056**	7	0.00	3	3	6	T3a	-	Low Risk	
**P5057**	10	0.48	4	5	9	T3b	+	High Risk	
**P5060**	4	0.02	3	4	7	T2	-	Low Risk	
**P5063**	12	0.02	4	5	9	T3b	-	High Risk	
**P5064**	16	0.00	4	3	7	T3a	+	High Risk	

**P5066**	13	0.00	4	3	7	T3b	+	High Risk	
**P5067**	10	0.09	4	3	7	T2c	+	Low Risk	
**P5068**	7	0.00	3	4	7	T2c	-	Low Risk	
**P5069**	5	0.00	4	3	7	T3a	+	High Risk	
**P5072**		0.06	4	3	7	T3b	+		*
**P5073**	5	0.88	3	3	6	T2	-	Low Risk	
**P5077**	14	2.94	4	5	9	T3b	-	High Risk	
**P5078**	20	0.00	4	5	9	T3b	+	High Risk	
**P5080**	7	0.27	3	4	7	T2	+	Low Risk	
**P5090**	11	0.00	3	4	7	T3b	-	High Risk	
**P5095**		0.00	3	4	7	T3a	-		*
**P5097**	14	0.31	5	4	9	T3b	+	High Risk	
**P5098**	13	0.44	4	3	7	T4	+	High Risk	
**P5099**	10	0.10	3	4	7	T3b	-	High Risk	
**P5101**		0.38	3	3	6	T2c	+	Low Risk	
**P5102**	6	0.00	4	5	9	T2c	+	High Risk	
**P5103**	16	0.18	4	3	7	T3a	+	High Risk	
**P5104**	7	0.00	3	4	7	T2c	-	Low Risk	
**P5105**	12	0.31	4	4	8	T2a	-	High Risk	
**P5106**	4	0.00	3	4	7	T2c	-	Low Risk	
**P5108**	9	3.80	4	5	9	T3a	+	High Risk	
**P5110**		0.00	3	4	7	T2	-	Low Risk	
**P5112**	5	0.03	4	4	8	T3a	+	High Risk	
**P5113**	9	0.52	3	4	7	T2a	-	Low Risk	
**P5115**	6	0.04	4	5	9	T3a	+	High Risk	
**P5116**	8	0.00	3	4	7	T2c	+	Low Risk	
**P5117**		0.99	3	4	7	T2	-	Low Risk	
**P5118**	6	0.00	4	3	7	T2c	+	Low Risk	
**P5119**	4	0.44	4	3	7	T2	-	Low Risk	
**P5120**	2	0.03	4	4	8	T3a	+	High Risk	
**P5121**	5	0.26	3	4	7	T2	+	Low Risk	
**P5122**	10	0.08	4	3	7	T3a	-	Low Risk	
**P5123**		0.18	4	3	7	T2c	+		*
**P5125**	6	0.27	4	3	7	T3a	-	Low Risk	

**P5126**	6	0.06	3	4	7	T3a	+	High Risk	
**P5127**	9	5.08	3	5	8	T2	-	High Risk	
**P5128**	12	0.14	3	4	7	T3a	+	High Risk	
**P5129**		1.72	3	4	7	T2	+	Low Risk	
**P5130**	5	0.00	4	5	9	T3b	+	High Risk	
**P5131**	11	0.66	4	3	7	T3a	+	High Risk	
**P5132**	5	0.14	3	4	7	T2	+	Low Risk	
**P5133**	4	0.15	4	3	7	T3a	-	Low Risk	
**P5135**	10	0.00	3	4	7	T3a	+	High Risk	
**P5137**	6	0.11	3	4	7	T2	-	Low Risk	
**P5139**	4	0.15	3	4	7	T3a	-	Low Risk	
							**High risk**	44	
							**Low risk**	31	
								75	

*** denotes insufficient RNA quality/quantity and sample excluded**
