# Supplementary material for: Circulating miRNAs as non-invasive biomarkers to predict aggressive prostate cancer after radical prostatectomy
Source: J Transl Med. 2019 May 23;17:173. doi: 10.1186/s12967-019-1920-5 (PMC6533745; doi:10.1186/s12967-019-1920-5)
Supplement: Supplementary file 2 — Additional file 2: Table S2. miRNA mimic sequences for control, miR-17, miR-20a, miR-20b and miR-106a that were used in in vitro validation studies. Sequences provided in 5’ – 3’ orientation. [file 12967_2019_1920_MOESM2_ESM.pdf]

miRNA mimic sequences

**Control**	F: UUCUCCGAACGUGUCACGUTT<br>R: ACGUGACACGUUCGGAGAATT
**miR-17**	F: CAAAGUGCUUACAGUGCAGGUAG<br>R: ACCUGCACUGUAAGCACUUUGUU
**miR-20a**	F: UAAAGUGCUUAUAGUGCAGGUAG<br>R: ACCUGCACUAUAAGCACUUUAUU
**miR-20b**	F:CAAAGUGCUCAUAGUGCAGGUAG<br>R:ACCUGCACUAUGAGCACUUUGUU
**miR-106a**	F: AAAAGUGCUUACAGUGCAGGUAG<br>R: ACCUGCACUGUAAGCACUUUUUU
