# Supplementary figures and images for: Circulating miRNAs as non-invasive biomarkers to predict aggressive prostate cancer after radical prostatectomy
Source: J Transl Med. 2019 May 23;17:173. doi: 10.1186/s12967-019-1920-5 (PMC6533745; doi:10.1186/s12967-019-1920-5)

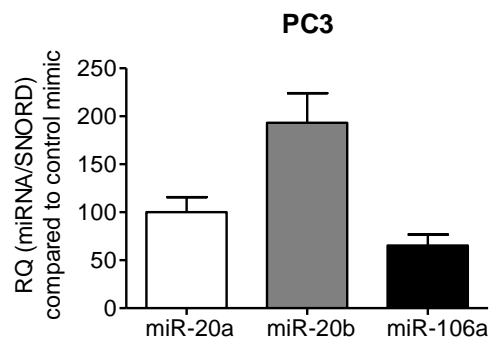

Supplement: Supplementary file 3 — Additional file 3: Figure S1. Representative qRT-PCR analysis for miR-20a, miR-20b and miR-106a expression in PC3 cells after miRNA mimic transfection. miRNA expression is normalized to endogenous control, SNORD. [file 12967_2019_1920_MOESM3_ESM.pdf]

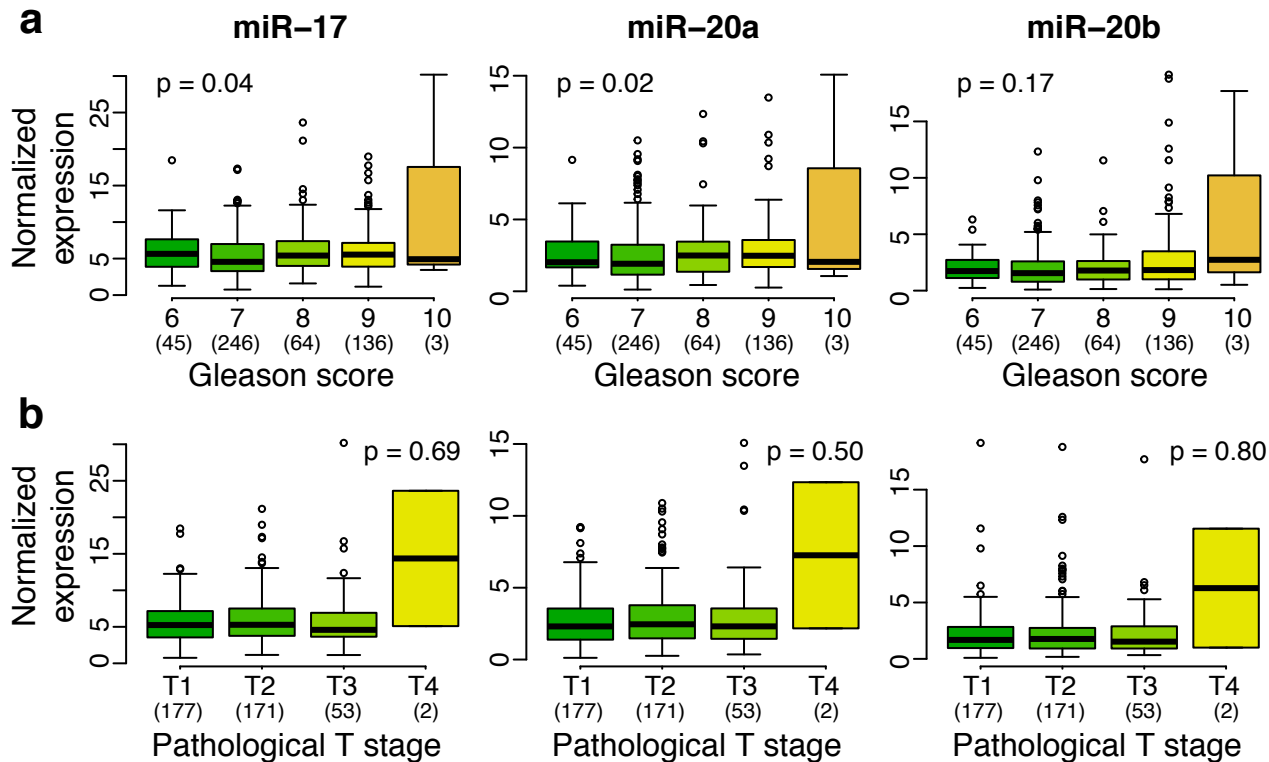

Supplement: Supplementary file 5 — Additional file 5: Figure S2. Expression of miR-17, miR-20a and miR-106a in patients with a Gleason score and b pathological T stage using the TCGA PRAD miRNA-seq dataset. Each box denotes the quartiles of the expression across samples. The black bars inside the boxes indicate the median expression of each miRNA. [file 12967_2019_1920_MOESM5_ESM.pdf]
