# Supplementary material for: Circulating miRNAs as non-invasive biomarkers to predict aggressive prostate cancer after radical prostatectomy
Source: J Transl Med. 2019 May 23;17:173. doi: 10.1186/s12967-019-1920-5 (PMC6533745; doi:10.1186/s12967-019-1920-5)
Supplement: Supplementary file 4 — Additional file 4: Table S3. Differential expression analysis of circulating serum miRNAs in low-risk vs high-risk patients. padj, p-value corrected for multiple testing using Bonferroni method. [file 12967_2019_1920_MOESM4_ESM.pdf]

id	baseMean	baseMeanA	baseMeanB	foldChange	log2FoldChange	pval	padj	minuslog2pval
**hsa-miR-1257**	21.125	18.87096774	22.82926829	1.209756098	0.274716211	0.73	0.919104	0.454031631
**hsa-miR-656-3p**	16.95833333	14.83870968	18.56097561	1.250848356	0.322906898	0.72	0.919104	0.473931188
**hsa-miR-574-5p**	104.9444444	77.35483871	125.804878	1.626334954	0.70162442	0.27	0.528701	1.888968688
**hsa-miR-503-5p**	11.875	11.87096774	11.87804878	1.000596501	0.000860312	1	1	0
**hsa-miR-188-5p**	26.05555556	19.35483871	31.12195122	1.60796748	0.685238229	0.3	0.569907	1.736965594
**hsa-miR-138-5p**	10.22222222	10.96774194	9.658536585	0.880631277	-0.18339001	0.82	0.955801	0.286304185
**hsa-miR-212-3p**	17.05555556	17.09677419	17.02439024	0.995766222	-0.006121017	0.97	0.996418	0.043943348
**hsa-miR-2053**	11.43055556	12.5483871	10.58536585	0.84356386	-0.245430807	0.79	0.954618	0.340075442
**hsa-miR-28-5p**	50.93055556	40.70967742	58.65853659	1.440899076	0.526969289	0.33	0.591941	1.59946207
**hsa-miR-1972**	53.22222222	48.80645161	56.56097561	1.158883175	0.212735138	0.7	0.919104	0.514573173
**hsa-miR-379-5p**	20.97222222	18.4516129	22.87804878	1.239894252	0.310217082	0.68	0.919104	0.556393349
**hsa-miR-30c-5p**	8.916666667	7.741935484	9.804878049	1.266463415	0.340805401	0.67	0.919104	0.577766999
**hsa-miR-96-5p**	12.125	12.09677419	12.14634146	1.004097561	0.005899452	1	1	0
**hsa-miR-1307-3p**	14.95833333	12.96774194	16.46341463	1.2695668	0.344336307	0.64	0.901814	0.64385619
**hsa-miR-423-3p**	23.61111111	17.25806452	28.41463415	1.646455437	0.719363464	0.24	0.515541	2.058893689
**hsa-miR-593-3p**	10.52777778	9.161290323	11.56097561	1.261937479	0.335640435	0.69	0.919104	0.535331733
**hsa-miR-584-5p**	7.222222222	8.806451613	6.024390244	0.684088269	-0.547745604	0.48	0.760035	1.058893689
**hsa-miR-127-3p**	22.66666667	23.80645161	21.80487805	0.915923062	-0.126701679	0.82	0.955801	0.286304185
**hsa-miR-575**	16.70833333	15.90322581	17.31707317	1.088903181	0.122875684	0.92	0.980533	0.120294234
**hsa-miR-125b-5p**	91.20833333	58.58064516	115.8780488	1.978094445	0.98411131	0.05	0.366548	4.321928095
**hsa-miR-216b-5p**	13.45833333	13.5483871	13.3902439	0.988327526	-0.016938873	0.96	0.996399	0.058893689
**hsa-miR-191-5p**	1269.819444	893.0322581	1554.707317	1.740930748	0.799858816	0.09	0.370329	3.473931188
**hsa-miR-29a-3p**	77.25	49.22580645	98.43902439	1.99974427	0.999815518	0.05	0.366548	4.321928095
**hsa-miR-181b-2-3p**	14.16666667	14.58064516	13.85365854	0.950140298	-0.073787537	0.91	0.980533	0.13606155
**hsa-miR-140-3p**	15.44444444	13.25806452	17.09756098	1.289597057	0.366920356	0.7	0.919104	0.514573173
**hsa-miR-149-5p**	91.19444444	66.51612903	109.8536585	1.651534149	0.7238068	0.24	0.515541	2.058893689
**hsa-miR-340-5p**	28.11111111	21.58064516	33.04878049	1.531408363	0.614859041	0.29	0.558245	1.785875195
**hsa-miR-548i**	9.430555556	8.451612903	10.17073171	1.203407187	0.267124878	0.81	0.954739	0.304006187
**hsa-miR-548ah-5p**	47.625	35.58064516	56.73170732	1.594454149	0.673062612	0.24	0.515541	2.058893689
**hsa-miR-374a-5p**	62.91666667	42.16129032	78.6097561	1.864500718	0.898789353	0.09	0.370329	3.473931188
**hsa-miR-651-5p**	18.52777778	17.35483871	19.41463415	1.118687098	0.161806564	0.87	0.961391	0.200912694
**hsa-miR-200c-3p**	10.55555556	10.29032258	10.75609756	1.045263399	0.063866538	0.96	0.996399	0.058893689
**hsa-miR-192-5p**	30.33333333	21	37.3902439	1.780487805	0.832272554	0.29	0.554246	1.785875195

**hsa-miR-3144-3p**	41.90277778	31.5483871	49.73170732	1.576362911	0.656599711	0.39	0.646249	1.358453971
**hsa-miR-6724-5p**	13.55555556	13.64516129	13.48780488	0.98846797	-0.016733877	0.96	0.996399	0.058893689
**hsa-miR-549a**	12.52777778	11.87096774	13.02439024	1.097163309	0.133778281	0.91	0.980533	0.13606155
**hsa-miR-1236-3p**	15.63888889	14.87096774	16.2195122	1.090683033	0.125231896	0.91	0.980533	0.13606155
**hsa-miR-139-3p**	16.09722222	14.25806452	17.48780488	1.226520252	0.294571055	0.73	0.919104	0.454031631
**hsa-let-7i-5p**	415.5277778	283.1935484	515.5853659	1.82061127	0.864422917	0.07	0.368445	3.836501268
**hsa-miR-644a**	16.02777778	14.06451613	17.51219512	1.245133139	0.316300015	0.73	0.919104	0.454031631
**hsa-let-7b-5p**	1443.222222	965.9677419	1804.073171	1.867632937	0.901210937	0.06	0.366548	4.058893689
**hsa-miR-141-3p**	10.31944444	11.51612903	9.414634146	0.817517251	-0.290678921	0.75	0.935622	0.415037499
**hsa-miR-873-3p**	57.58333333	52.32258065	61.56097561	1.176566118	0.234582397	0.8	0.954618	0.321928095
**hsa-miR-155-5p**	65.94444444	42.19354839	83.90243902	1.988513463	0.99169033	0.06	0.366548	4.058893689
**hsa-miR-941**	9.236111111	9.774193548	8.829268293	0.903324479	-0.146683791	0.83	0.956438	0.268816758
**hsa-miR-891b**	21.22222222	22.35483871	20.36585366	0.911026643	-0.134434849	0.84	0.960795	0.251538767
**hsa-miR-142-3p**	464.3611111	325.2580645	569.5365854	1.751029867	0.808203692	0.09	0.370329	3.473931188
**hsa-let-7c-5p**	100.3472222	70.16129032	123.1707317	1.755536866	0.811912293	0.11	0.370329	3.184424571
**hsa-miR-217**	11.25	12.70967742	10.14634146	0.798316207	-0.324967796	0.71	0.919104	0.49410907
**hsa-miR-450a-5p**	27.52777778	19.09677419	33.90243902	1.775296638	0.828060108	0.16	0.425878	2.64385619
**hsa-miR-30b-5p**	14.83333333	12.87096774	16.31707317	1.267742527	0.34226177	0.63	0.896521	0.666576266
**hsa-miR-592**	13.97222222	15.96774194	12.46341463	0.780537078	-0.357460928	0.71	0.919104	0.49410907
**hsa-miR-1287-5p**	13.97222222	13.29032258	14.48780488	1.090101823	0.124462899	0.92	0.980533	0.120294234
**hsa-miR-590-5p**	19.51388889	21.06451613	18.34146341	0.870727972	-0.199706024	0.77	0.947173	0.377069649
**hsa-miR-1226-3p**	12.09722222	13.48387097	11.04878049	0.819407165	-0.287347586	0.76	0.940738	0.395928676
**hsa-miR-34c-3p**	9.736111111	9.548387097	9.87804878	1.034525379	0.048969038	0.99	1	0.01449957
**hsa-miR-424-5p**	18.375	18.90322581	17.97560976	0.950928161	-0.07259174	0.9	0.980533	0.152003093
**hsa-miR-520a-5p**	14.81944444	12.12903226	16.85365854	1.389530358	0.474597354	0.63	0.896521	0.666576266
**hsa-miR-640**	17.86111111	17.48387097	18.14634146	1.037890379	0.053654076	0.97	0.996418	0.043943348
**hsa-miR-877-5p**	20.51388889	14.58064516	25	1.71460177	0.777873538	0.32	0.591941	1.64385619
**hsa-miR-515-3p**	12.59722222	13.25806452	12.09756098	0.912468103	-0.132153968	0.87	0.961391	0.200912694
**hsa-miR-302c-3p**	13.02777778	11.67741935	14.04878049	1.203072362	0.26672342	0.8	0.954618	0.321928095
**hsa-miR-21-5p**	307.2916667	194.3548387	392.6829268	2.020443275	1.014671848	0.04	0.366548	4.64385619
**hsa-miR-4531**	16.84722222	14.70967742	18.46341463	1.255188276	0.327903782	0.64	0.901814	0.64385619
**hsa-miR-665**	12.55555556	15.83870968	10.07317073	0.635984303	-0.652936937	0.5	0.773139	1
**hsa-miR-4443**	31.63888889	30.12903226	32.7804878	1.088003343	0.121682989	0.89	0.980533	0.168122759
**hsa-miR-628-3p**	8.25	8.032258065	8.414634146	1.047605054	0.067094925	0.95	0.996399	0.074000581

**hsa-miR-548al**	14.29166667	12.87096774	15.36585366	1.193838254	0.255607388	0.8	0.954618	0.321928095
**hsa-let-7a-5p**	1612.291667	1291.516129	1854.829268	1.436164231	0.522220736	0.27	0.528701	1.888968688
**hsa-miR-1246**	203.7083333	296	133.9268293	0.452455504	-1.144152174	0.3	0.558245	1.736965594
**hsa-miR-1827**	6.402777778	7.064516129	5.902439024	0.835505067	-0.259279517	0.74	0.926468	0.434402824
**hsa-miR-423-5p**	405.625	276.0322581	503.6097561	1.824459792	0.867469357	0.07	0.368445	3.836501268
**hsa-miR-4536-5p**	18.15277778	16.77419355	19.19512195	1.144324578	0.194496318	0.79	0.954618	0.340075442
**hsa-miR-2682-5p**	64.79166667	46.80645161	78.3902439	1.674774336	0.743966715	0.2	0.479635	2.321928095
**hsa-miR-4787-5p**	16.04166667	14.87096774	16.92682927	1.138246654	0.186813218	0.84	0.960795	0.251538767
**hsa-miR-1268a**	10.55555556	9.064516129	11.68292683	1.288863814	0.366099831	0.66	0.913145	0.59946207
**hsa-miR-19a-3p**	66.83333333	37.5483871	88.97560976	2.369625346	1.244658977	0.03	0.366548	5.058893689
**hsa-miR-122-5p**	2463.888889	1632.774194	3092.292683	1.893888754	0.92135159	0.05	0.366548	4.321928095
**hsa-miR-382-5p**	45.23611111	42.38709677	47.3902439	1.118034673	0.160964931	0.78	0.947173	0.358453971
**hsa-miR-577**	15.29166667	12.25806452	17.58536585	1.434595635	0.520644147	0.57	0.851726	0.810966176
**hsa-miR-140-5p**	56.15277778	37.5483871	70.2195122	1.870107284	0.903121036	0.09	0.370329	3.473931188
**hsa-miR-6721-5p**	26.84722222	24.29032258	28.7804878	1.18485408	0.244709395	0.85	0.961391	0.234465254
**hsa-miR-148a-3p**	286.2777778	207.3225806	345.9756098	1.668779198	0.738793079	0.13	0.391186	2.943416472
**hsa-miR-216a-5p**	9.805555556	8.580645161	10.73170732	1.250687695	0.322721584	0.72	0.919104	0.473931188
**hsa-miR-302f**	10.04166667	11.22580645	9.146341463	0.814760303	-0.295552405	0.72	0.919104	0.473931188
**hsa-miR-324-5p**	37.69444444	20.4516129	50.73170732	2.48057244	1.310673089	0.02	0.366548	5.64385619
**hsa-miR-518b**	8.055555556	6.290322581	9.390243902	1.492808005	0.578028628	0.48	0.760035	1.058893689
**hsa-miR-520f-3p**	38.38888889	26.61290323	47.29268293	1.777058389	0.829491085	0.35	0.601768	1.514573173
**hsa-miR-151a-3p**	46.08333333	27.19354839	60.36585366	2.219859387	1.150468295	0.04	0.366548	4.64385619
**hsa-miR-15a-5p**	139.875	92.29032258	175.8536585	1.905439851	0.930124068	0.06	0.366548	4.058893689
**hsa-miR-4454+hsa-miR-7975**	2244.930556	1964.548387	2456.926829	1.250631873	0.322657191	0.5	0.773139	1
**hsa-miR-1180-3p**	15.72222222	9.451612903	20.46341463	2.165071173	1.114414452	0.1	0.370329	3.321928095
**hsa-miR-128-1-5p**	18.26388889	10.80645161	23.90243902	2.211867492	1.145264959	0.25	0.515541	2
**hsa-miR-374b-5p**	14.70833333	9.64516129	18.53658537	1.921853332	0.94249824	0.16	0.425878	2.64385619
**hsa-miR-92a-3p**	99.33333333	62.32258065	127.3170732	2.042872292	1.030599018	0.04	0.366548	4.64385619
**hsa-miR-98-5p**	61.34722222	49.19354839	70.53658537	1.433858457	0.519902615	0.32	0.591941	1.64385619
**hsa-miR-24-3p**	177.875	117.5483871	223.4878049	1.901240931	0.926941366	0.06	0.366548	4.058893689
**hsa-miR-505-3p**	6.958333333	3.903225806	9.268292683	2.374521266	1.247636677	0.15	0.414946	2.736965594
**hsa-miR-150-5p**	885.8888889	658.7419355	1057.634146	1.605536386	0.683055361	0.15	0.418199	2.736965594
**hsa-miR-19b-3p**	244.0833333	138.0967742	324.2195122	2.347770352	1.231291298	0.02	0.366548	5.64385619

hsa-miR-193a-5p+hsa-miR-193b-5p	14.56944444	11.06451613	17.2195122	1.556282443	0.638103913	0.35	0.601768	1.514573173
hsa-miR-548n	8.708333333	3.870967742	12.36585366	3.194512195	1.675595647	0.05	0.366548	4.321928095
hsa-miR-29b-3p	13.06944444	10.03225806	15.36585366	1.531644577	0.615081554	0.38	0.639909	1.395928676
hsa-miR-93-5p	1065.444444	678.3225806	1358.146341	2.002213077	1.001595515	0.04	0.366548	4.64385619
hsa-miR-509-5p	3.75	3.967741935	3.585365854	0.903628792	-0.146197855	0.87	0.961391	0.200912694
hsa-miR-20a-5p+hsa-miR-20b-5p	291.125	165.0645161	386.4390244	2.341139292	1.227210774	0.01	0.366548	6.64385619
hsa-miR-323b-3p	8.958333333	7.548387097	10.02439024	1.328017511	0.40927417	0.61	0.878678	0.713118852
hsa-miR-1285-3p	6.222222222	4.451612903	7.56097561	1.698480028	0.764244254	0.39	0.646249	1.358453971
hsa-miR-221-3p	154.25	93.38709677	200.2682927	2.144496398	1.100638893	0.03	0.366548	5.058893689
hsa-miR-376a-3p	37.65277778	25.93548387	46.51219512	1.793380658	0.842681743	0.13	0.400889	2.943416472
hsa-miR-526a+hsa-miR-518c-5p+hsa-miR-518d-5p	18.43055556	11.74193548	23.48780488	2.000335031	1.000241653	0.16	0.425878	2.64385619
hsa-let-7d-5p	390.2777778	280.7741935	473.0731707	1.684888361	0.752653003	0.12	0.378233	3.058893689
hsa-miR-196a-5p	20.88888889	18.19354839	22.92682927	1.260162602	0.3336099	0.6	0.877303	0.736965594
hsa-miR-1299	27.52777778	17.80645161	34.87804878	1.958731	0.969919281	0.11	0.370329	3.184424571
hsa-miR-506-3p	13.06944444	8.870967742	16.24390244	1.83113082	0.872734864	0.21	0.493144	2.251538767
hsa-miR-491-5p	10.20833333	6.129032258	13.29268293	2.168806162	1.116901117	0.14	0.408613	2.836501268
hsa-miR-513b-5p	10.63888889	5.709677419	14.36585366	2.516053466	1.33116258	0.08	0.368445	3.64385619
hsa-miR-514a-5p	9.625	6.129032258	12.26829268	2.001668806	1.001203287	0.19	0.467373	2.395928676
hsa-miR-1183	20.26388889	10.25806452	27.82926829	2.712916091	1.439844427	0.04	0.366548	4.64385619
hsa-miR-1910-5p	27.75	17.19354839	35.73170732	2.078204366	1.055337532	0.08	0.368445	3.64385619
hsa-miR-451a	73635.125	44574.29032	95607.95122	2.144912471	1.100918776	0.04	0.366548	4.64385619
hsa-miR-144-3p	206.3611111	144.6129032	253.0487805	1.749835422	0.807219238	0.1	0.370329	3.321928095
hsa-miR-4421	5.069444444	2.161290323	7.268292683	3.362941391	1.749723636	0.08	0.368445	3.64385619
hsa-miR-532-5p	8.083333333	4.193548387	11.02439024	2.628893058	1.394455455	0.09	0.370329	3.473931188
hsa-miR-320e	1701.333333	986.4193548	2241.878049	2.27274337	1.184434789	0.01	0.366548	6.64385619
hsa-miR-29c-3p	23.48611111	13.4516129	31.07317073	2.309995906	1.207890295	0.05	0.366548	4.321928095
hsa-miR-145-5p	9.805555556	6.193548387	12.53658537	2.024136179	1.017306354	0.18	0.454091	2.473931188
hsa-miR-342-3p	179.5833333	125.3548387	220.5853659	1.759687684	0.815319397	0.1	0.370329	3.321928095

**hsa-miR-425-5p**	59.81944444	34.19354839	79.19512195	2.316083755	1.211687426	0.02	0.366548	5.64385619
**hsa-miR-612**	103.5555556	86.09677419	116.7560976	1.356103044	0.439466806	0.38	0.643574	1.395928676
**hsa-miR-1306-5p**	2.583333333	1.064516129	3.731707317	3.505543237	1.809638029	0.18	0.446603	2.473931188
**hsa-miR-23b-3p**	27.73611111	19	34.34146341	1.807445443	0.853952101	0.15	0.414946	2.736965594
**hsa-miR-28-3p**	6.763888889	4.870967742	8.195121951	1.682442255	0.750556989	0.39	0.644904	1.358453971
**hsa-miR-585-3p**	13.90277778	12.58064516	14.90243902	1.184552846	0.244342562	0.73	0.919104	0.454031631
**hsa-miR-185-5p**	205.3888889	117.1935484	272.0731707	2.321571234	1.215101548	0.02	0.366548	5.64385619
**hsa-miR-497-5p**	5.013888889	2.419354839	6.975609756	2.883252033	1.527696952	0.12	0.382728	3.058893689
**hsa-miR-548a-5p**	4.847222222	3.193548387	6.097560976	1.909337275	0.93307197	0.35	0.601768	1.514573173
**hsa-miR-582-5p**	7.236111111	6.322580645	7.926829268	1.253733201	0.32623037	0.71	0.919104	0.49410907
**hsa-miR-626**	8.486111111	6.580645161	9.926829268	1.508488761	0.593103948	0.46	0.737438	1.120294234
**hsa-miR-132-3p**	10.56944444	7.903225806	12.58536585	1.592434047	0.671233622	0.37	0.629888	1.434402824
**hsa-miR-18a-5p**	32.08333333	17.22580645	43.31707317	2.514661551	1.33036424	0.02	0.366548	5.64385619
**hsa-miR-126-3p**	1355.361111	962.3225806	1652.536585	1.717237669	0.780089725	0.1	0.370329	3.321928095
**hsa-miR-548g-3p**	8.597222222	8.451612903	8.707317073	1.030255074	0.043001568	0.98	0.996877	0.029146346
**hsa-miR-598-3p**	33.19444444	27.03225806	37.85365854	1.400314337	0.485750714	0.57	0.852536	0.810966176
**hsa-miR-574-3p**	5.888888889	5.032258065	6.536585366	1.298936836	0.377331277	0.69	0.919104	0.535331733
**hsa-miR-642a-5p**	3.277777778	3.387096774	3.195121951	0.943321719	-0.08417821	0.92	0.980533	0.120294234
**hsa-miR-301a-3p**	9.25	6.129032258	11.6097561	1.894223363	0.921606461	0.24	0.515541	2.058893689
**hsa-miR-26b-5p**	287.3333333	168.1612903	377.4390244	2.244505996	1.16639795	0.05	0.366548	4.321928095
**hsa-miR-378i**	37.54166667	26.70967742	45.73170732	1.712177448	0.775832229	0.17	0.425878	2.556393349
**hsa-miR-4286**	140.5277778	124.8709677	152.3658537	1.220186376	0.287101528	0.57	0.851726	0.810966176
**hsa-miR-4516**	65.25	33.64516129	89.14634146	2.649603629	1.405776554	0.11	0.377989	3.184424571
**hsa-miR-421**	8.902777778	5.935483871	11.14634146	1.877916225	0.909132705	0.25	0.515541	2
**hsa-miR-543**	9.097222222	7.64516129	10.19512195	1.333539158	0.415260189	0.6	0.877303	0.736965594
**hsa-miR-324-3p**	5.291666667	3.64516129	6.536585366	1.793222534	0.842554534	0.38	0.639909	1.395928676
**hsa-miR-365a-3p+hsa-miR-365b-3p**	6.138888889	5	7	1.4	0.485426827	0.6	0.877303	0.736965594
**hsa-miR-1285-5p**	17.875	12.80645161	21.70731707	1.695029797	0.761310634	0.24	0.515541	2.058893689
**hsa-miR-183-5p**	6.722222222	5.903225806	7.341463415	1.243635879	0.314564144	0.73	0.919104	0.454031631
**hsa-miR-10a-5p**	10.15277778	6.935483871	12.58536585	1.814634146	0.859678712	0.25	0.515541	2
**hsa-miR-1290**	36.90277778	22.77419355	47.58536585	2.08944241	1.063117995	0.06	0.366548	4.058893689
**hsa-miR-107**	111.875	70.38709677	143.2439024	2.035087523	1.025090842	0.04	0.366548	4.64385619

**hsa-miR-34a-5p**	13.73611111	9.967741935	16.58536585	1.663904018	0.734572214	0.29	0.554246	1.785875195
**hsa-miR-301a-5p**	8.569444444	4.451612903	11.68292683	2.624425592	1.392001695	0.14	0.408613	2.836501268
**hsa-miR-199b-5p**	6.916666667	4.516129032	8.731707317	1.933449477	0.951177066	0.27	0.528701	1.888968688
**hsa-let-7g-5p**	441.125	312.6129032	538.2926829	1.721914474	0.784013487	0.1	0.370329	3.321928095
**hsa-miR-26a-5p**	219.7222222	155.9677419	267.9268293	1.717834893	0.780591381	0.11	0.370329	3.184424571
**hsa-miR-888-5p**	7.722222222	7.193548387	8.12195122	1.129060483	0.175122773	0.85	0.961391	0.234465254
**hsa-miR-186-5p**	18.66666667	11.19354839	24.31707317	2.172418641	1.119302148	0.08	0.368445	3.64385619
**hsa-miR-10b-5p**	10.875	6.870967742	13.90243902	2.02335967	1.016752795	0.17	0.429534	2.556393349
**hsa-miR-197-5p**	8.152777778	6.580645161	9.341463415	1.419536107	0.505419546	0.54	0.824472	0.888968688
**hsa-miR-301b-3p**	4.722222222	4.677419355	4.756097561	1.016820858	0.02406553	1	1	0
**hsa-miR-1255a**	8.805555556	5.870967742	11.02439024	1.877780756	0.909028628	0.25	0.515541	2
**hsa-miR-1295a**	13.25	12.41935484	13.87804878	1.117453278	0.160214512	0.83	0.956438	0.268816758
**hsa-miR-23a-3p**	2072.944444	1557	2463.04878	1.581919576	0.661676256	0.16	0.425878	2.64385619
**hsa-miR-30e-5p**	91.90277778	56.29032258	118.8292683	2.111007058	1.077931403	0.04	0.366548	4.64385619
**hsa-miR-335-5p**	7.861111111	5.129032258	9.926829268	1.935419543	0.952646335	0.25	0.515541	2
**hsa-miR-514b-5p**	20.18055556	13.93548387	24.90243902	1.786980578	0.837523954	0.27	0.528701	1.888968688
**hsa-miR-199a-3p+hsa-miR-199b-3p**	834.2638889	597.8064516	1013.04878	1.694609982	0.760953273	0.11	0.370329	3.184424571
**hsa-miR-1537-3p**	11.33333333	6.870967742	14.70731707	2.140501546	1.097948877	0.17	0.425878	2.556393349
**hsa-miR-3168**	5.208333333	3.709677419	6.341463415	1.709437964	0.773522068	0.42	0.682324	1.251538767
**hsa-miR-664a-3p**	8.611111111	5.35483871	11.07317073	2.067881281	1.048153362	0.19	0.463572	2.395928676
**hsa-miR-337-3p**	18.01388889	13.58064516	21.36585366	1.573257633	0.653754942	0.34	0.601768	1.556393349
**hsa-miR-363-3p**	38.70833333	28.22580645	46.63414634	1.652181185	0.724371907	0.21	0.490084	2.251538767
**hsa-miR-199a-5p**	49.19444444	33.29032258	61.2195122	1.838958215	0.878888699	0.1	0.370329	3.321928095
**hsa-miR-16-5p**	7170.819444	4387.870968	9275	2.113781392	1.07982618	0.04	0.366548	4.64385619
**hsa-miR-299-3p**	4.805555556	3.322580645	5.926829268	1.783802984	0.834956282	0.4	0.654271	1.321928095
**hsa-miR-454-3p**	14.20833333	9.677419355	17.63414634	1.822195122	0.865677452	0.21	0.487612	2.251538767
**hsa-miR-660-5p**	30.63888889	17.67741935	40.43902439	2.287609044	1.193840514	0.08	0.368445	3.64385619
**hsa-miR-27b-3p**	34.83333333	21.96774194	44.56097561	2.028473192	1.020394236	0.07	0.368445	3.836501268
**hsa-miR-106b-5p**	25.36111111	14.29032258	33.73170732	2.360458074	1.239066858	0.04	0.366548	4.64385619
**hsa-miR-625-5p**	4.986111111	4	5.731707317	1.432926829	0.518964942	0.6	0.877303	0.736965594
**hsa-miR-130a-3p**	293.1527778	178.9677419	379.4878049	2.12042573	1.084353952	0.03	0.366548	5.058893689
**hsa-miR-15b-5p**	530.9305556	371.9677419	651.1219512	1.750479619	0.807750265	0.09	0.370329	3.473931188

**hsa-miR-222-3p**	136.2083333	92.09677419	169.5609756	1.841117423	0.880581642	0.08	0.368445	3.64385619
**hsa-miR-25-3p**	2834.944444	1968.451613	3490.097561	1.773016689	0.826206116	0.08	0.368445	3.64385619
**hsa-miR-769-5p**	9.125	9.548387097	8.804878049	0.922132498	-0.116954033	0.86	0.961391	0.217591435
**hsa-miR-146a-5p**	289.4583333	194.1612903	361.5121951	1.861916938	0.896788714	0.07	0.368445	3.836501268
**hsa-let-7e-5p**	23.80555556	18.51612903	27.80487805	1.501657177	0.586555488	0.33	0.597094	1.59946207
**hsa-miR-361-3p**	7.472222222	5.290322581	9.12195122	1.724271267	0.785986761	0.35	0.601768	1.514573173
**hsa-miR-1322**	8.888888889	7.161290323	10.19512195	1.423643155	0.509587571	0.52	0.805131	0.943416472
**hsa-miR-22-3p**	276.9166667	175.2258065	353.804878	2.019136822	1.013738675	0.04	0.366548	4.64385619
**hsa-miR-106a-5p+hsa-miR-17-5p**	146.9722222	79.64516129	197.8780488	2.48449555	1.312952958	0.01	0.366548	6.64385619
**hsa-miR-181a-5p**	118.0972222	85.51612903	142.7317073	1.669061836	0.739037405	0.14	0.408613	2.836501268
**hsa-miR-148b-3p**	74.34722222	47.80645161	94.41463415	1.974934992	0.981805166	0.06	0.366548	4.058893689
**hsa-miR-223-3p**	7873.986111	5219.451613	9881.073171	1.893124777	0.920769503	0.05	0.366548	4.321928095
**hsa-miR-652-3p**	13.11111111	6.612903226	18.02439024	2.7256395	1.44659476	0.04	0.366548	4.64385619
**hsa-miR-630**	23.04166667	11.67741935	31.63414634	2.709001482	1.437761183	0.12	0.382728	3.058893689
**hsa-miR-197-3p**	6.458333333	6.258064516	6.609756098	1.056198139	0.078880505	0.95	0.996399	0.074000581
**hsa-miR-495-3p**	16.5	14.51612903	18	1.24	0.310340121	0.64	0.901814	0.64385619
**hsa-miR-432-5p**	7.736111111	7.129032258	8.195121951	1.149541993	0.201059169	0.82	0.956438	0.286304185
**hsa-miR-486-3p**	61.22222222	42.4516129	75.41463415	1.776484543	0.829025136	0.12	0.378233	3.058893689
**hsa-miR-4455**	5.152777778	2.225806452	7.365853659	3.309296571	1.726524588	0.11	0.370329	3.184424571
**hsa-let-7f-5p**	80.08333333	61.87096774	93.85365854	1.516925659	0.601150385	0.24	0.515541	2.058893689
**hsa-miR-7-5p**	58.11111111	35.32258065	75.34146341	2.132954672	1.092853307	0.04	0.366548	4.64385619
**hsa-miR-361-5p**	40.97222222	31.06451613	48.46341463	1.560089152	0.641628475	0.25	0.515541	2
**hsa-miR-30d-5p**	252.9861111	165.0645161	319.4634146	1.935385158	0.952620703	0.05	0.366548	4.321928095
**hsa-miR-99b-5p**	16.875	14.29032258	18.82926829	1.317623741	0.397938454	0.55	0.831773	0.862496476
**hsa-miR-206**	8.597222222	8.35483871	8.780487805	1.050946417	0.071689114	0.95	0.996399	0.074000581
**hsa-miR-125a-5p**	36.36111111	27.19354839	43.29268293	1.592020369	0.670858794	0.23	0.515541	2.120294234
**hsa-miR-629-5p**	5.361111111	3.516129032	6.756097561	1.921458939	0.942202147	0.32	0.591941	1.64385619
**hsa-miR-3065-5p**	94.26388889	68.06451613	114.0731707	1.675956537	0.744984736	0.24	0.515541	2.058893689
**hsa-miR-30a-5p**	21.75	14.51612903	27.2195122	1.875121951	0.906984426	0.14	0.408613	2.836501268
